# COVID-19 and the Gastrointestinal System: What Trainees Need to Know

**DOI:** 10.14309/crj.0000000000000391

**Published:** 2020-06-12

**Authors:** Ahmad Najdat Bazarbashi, C. Roberto Simons-Linares

**Affiliations:** 1 Division of Gastroenterology, Hepatology and Endoscopy, Brigham and Women's Hospital, Harvard Medical School, Boston, MA; 2 Gastroenterology and Hepatology Department, Digestive Disease Institute, Cleveland Clinic Foundation, Cleveland, OH

## INTRODUCTION

Severe acute respiratory syndrome coronavirus 2 (SARS-CoV-2), also called 2019-nCoV, is a novel coronavirus that broke out in Wuhan, China, in December 2019. This contagious virus, which causes coronavirus disease 2019 or COVID-19, has now spread worldwide causing disruptions in April 2020 activities and yielding a significant global public health concern. To date, there have been more than 1,000,000 cases worldwide with more than 50,000 deaths and COVID-19 was declared a pandemic by the World Health Organization on March 11, 2020.^1,2^ In the United States, more than 1 million individuals have been tested thus far. The total number of individuals in the United States who have tested positive has exceeded 215,000 with more than 5,000 deaths related to COVID-19 documented to date.^2,3^ The main mode of transmission is through respiratory droplets and contact routes with a mean incubation period of 5.1 days.^4^ Although some patients may remain asymptomatic, common symptoms of COVID-19 include fever, cough, shortness of breath, muscle ache, and sore throat.^5,6^ Viral pneumonia and acute respiratory distress syndrome can occur and result in intensive care admission and ventilatory support in up to 20% of patients.^5,6^ Diagnosis entails real-time reverse transcriptase-polymerase chain reaction of samples obtained through nasopharyngeal or oral swabs. Cross sectional imaging of the chest is commonly performed for those with a concern for pulmonary disease to evaluate for grand glass opacities. Although there have been promising breakthroughs with various medications to treat COVID-19, there still remains a significant need for trials and studies to elucidate best practices and therapeutic options. To date, hydroxychloroquine, remdesivir, protease inhibitors, human monoclonal antibodies, and other therapies have all been implicated as possible emerging therapies.^7^ Although symptomatic COVID-19 often presents with fever and respiratory symptoms, there is now emerging data on gastrointestinal (GI) manifestations of this disease. This commentary will focus on COVID-19 and the GI system, including GI presentation, viral stool shedding, and endoscopic considerations.

## GASTROINTESTINAL AND LIVER INVOLVEMENT WITH COVID-19

It is postulated that SARS-CoV-2 can bind to the angiotensin converting enzyme 2 receptors in the host. Angiotensin converting enzyme 2 is expressed in the intestinal tract and is an important regulator of intestinal inflammation.^8^ Digestive symptoms are therefore not uncommon in COVID-19, with emerging literature confirming this.

A recent cross-sectional multicenter study from Hubei, China, including 204 patients with COVID-19 revealed that despite fever or respiratory symptoms being the most common presentation, half of the patients reported digestive symptoms. These included, in order of frequency, anorexia, diarrhea, vomiting, and abdominal pain.^9^ Patients with digestive symptoms had longer time from onset to admission, with digestive symptoms becoming more pronounced as disease severity increased.^9^ Another study from China including 206 patients with low severity COVID-19 revealed that 48 patients had digestive symptoms alone, whereas 69 patients had combination of respiratory and digestive symptoms. Of those, 32% presented with diarrhea, which was the first symptom in approximately 20% of patients.^10^ More recently, in a study including 254 patients with novel coronavirus-infected pneumonia, 66 (26%) complained of GI symptoms. Gastrointestinal symptoms were found to be significantly higher in female patients than male patients. Sore throat, dizziness, and fatigue were also more common in patients with GI symptoms. In addition, those with GI symptoms had lower hemoglobin but higher C-reactive protein and alanine aminotransferase than those without GI symptoms.^11^ Outside of Wuhan, one study from China of 651 patients revealed that 74 (11.1%) presented with at least one GI symptom.^12^

Liver injury from COVID-19 has also been reported. This is speculated to occur from direct viral infection and injury to hepatocytes. Large scale studies reveal that 14%–53% of patients with COVID-19 had elevations in aspartate aminotransferase and alanine aminotransferase.^13^ In addition, higher rates of liver dysfunction have been observed in patients with more severe disease and those requiring intensive care unit.^14,15^

It is imperative for trainees and physicians to recognize and appreciate the rate of GI symptoms and liver abnormalities in patients with COVID-19 and to have a low threshold to test for SARS-CoV-2 in patients with GI symptoms and high clinical suspicion or risk factors for COVID-19.

## VIRAL FECAL SHEDDING AND RISK OF FECAL-ORAL TRANSMISSION

Although observational studies have emerged on COVID-19 causing GI symptoms, there have also emerging data from several case series on the presence of viral RNA in stool. Although there have been no documented cases of fecal-oral route transmission of SARS-CoV-2, studies and findings have sparked debate on whether persistent fecal viral shedding can lead to fecal-oral transmission.^16^ In the United States, the first case of COVID-19, published in the New England Journal of Medicine, reported loose stools for which a stool sample sent for real-time reverse transcriptase-polymerase chain reaction was positive.^17^ A recent study evaluating SARS-CoV-2 in difference types of clinical specimens included 1,070 various samples obtained from 205 patients with COVID-19. The results revealed that bronchoalveolar lavage had the highest positive rates (93%). Feces showed positive results in 29% of patients.^18^ Another study of 73 SARS-CoV-2-infected patients revealed that 39 (53.4%) tested positive for SARS-CoV-2 RNA in stool. The duration time of positive stool ranged from 1 to 12 days. Importantly, 17 patients (23.3%) remained positive in stool even after testing negative in respiratory samples.^19^ Data on prolonged and extended duration of viral shedding, weeks after respiratory samples test negative, have also been published.^20^ In addition, a recent study of 9 patients from Germany showed that actual virus isolation from stool samples was never achieved, regardless of viral RNA concentration detected in stool. Of note, stool and sputum samples remained RNA-positive for the virus >3 weeks in 66% of patients even when the patients completely recovered and were asymptomatic.^21^

It is imperative for trainees to appreciate that fecal viral shedding has been observed in patients with COVID-19 and can be prolonged, extending beyond clearance of respiratory samples. This may have future implications for fecal sample testing, timing, and the type of the testing, particularly in those with cleared respiratory samples. Detecting live virus in stool and the possibility of fecal-oral transmission remains to be investigated.

### Endoscopy in patients with COVID-19

Data remain limited on endoscopic finding in patients with COVID-19, particularly given the low indication and high threshold needed for endoscopy in these patients. However, a recent case report from Michigan confirmed hemorrhagic colitis on colonoscopy in a 71-year-old patient returning from Egypt who was later diagnosed with COVID-19. This case does support the hypothesis of SARS-CoV-2 causing digestive symptoms. Although this is the only case report of endoscopic findings in patients with COVID-19, we anticipate more data to emerge on endoscopic findings in patients later being confirmed to have COVID-19.

It is important to note and highlight the importance of safe endoscopic practices in patients with suspected or confirmed COVID-19. Repici et al have highlighted the practices that need to be considered in the endoscopy unit such as dress code and personal protective equipment, use of negative pressure rooms, reprocessing of flexible endoscopes, and decontamination policies.^22^ Finally, a joint statement from the multiple gastroenterology and liver societies in the United States has been released to help and educate GI practitioners.^23^ The recommendations include considering rescheduling elective nonurgent endoscopic procedures, prescreen all patients for high risk exposures, ensure personal protective equipement is available and worn by all endoscopy team members (including how to don and doff), isolation precautions for patients with COVID-19, and others.^23^ More recently, the American Gastroenterology Association Institute has also released recommendations for GI procedures in which they recommend the use of N-95 (or N-99, or powered air-purifying respirator) masks instead of surgical masks.^24^

We hope this short communication helps trainees navigate the rapidly growing COVID-19 literature focusing on the aforementioned topics. It is imperative that trainees review published guidelines from GI and liver societies and remain up to date with the rapidly changing landscape, particularly to changes that apply to endoscopy practices.

## DISCLOSURES

Author contributions: AN Bazarbashi and CR Simons-Linares contributed equally to this manuscript. CR Simons-Linares is the article guarantor.

Financial disclosure: None to report.
